# Annexin A1 is involved in the acquisition and maintenance of a stem cell-like/aggressive phenotype in prostate cancer cells with acquired resistance to zoledronic acid

**DOI:** 10.18632/oncotarget.4725

**Published:** 2015-07-28

**Authors:** Valentina Bizzarro, Raffaella Belvedere, Maria Rita Milone, Biagio Pucci, Rita Lombardi, Francesca Bruzzese, Ada Popolo, Luca Parente, Alfredo Budillon, Antonello Petrella

**Affiliations:** ^1^ Department of Pharmacy, University of Salerno, Fisciano (SA), Italy; ^2^ Centro Ricerche Oncologiche Mercogliano, Istituto Nazionale Tumori Fondazione G. Pascale – IRCCS, Naples, Italy; ^3^ Experimental Pharmacology Unit, Istituto Nazionale Tumori Fondazione G. Pascale – IRCCS, Naples, Italy

**Keywords:** annexin A1, prostate cancer, cell invasion, EMT, cancer stem cells

## Abstract

In this study, we have characterized the role of annexin A1 (ANXA1) in the acquisition and maintenance of stem-like/aggressive features in prostate cancer (PCa) cells comparing zoledronic acid (ZA)-resistant DU145R80 with their parental DU145 cells. ANXA1 is over-expressed in DU145R80 cells and its down-regulation abolishes their resistance to ZA. Moreover, ANXA1 induces DU145 and DU145R80 invasiveness acting through formyl peptide receptors (FPRs). Also, ANXA1 knockdown is able to inhibit epithelial to mesenchymal transition (EMT) and to reduce focal adhesion kinase (FAK) and metalloproteases (MMP)-2/9 expression in PCa cells. DU145R80 show a cancer stem cell (CSC)-like signature with a high expression of CSC markers including CD44, CD133, NANOG, Snail, Oct4 and ALDH7A1 and CSC-related genes as STAT3. Interestingly, ANXA1 knockdown induces these cells to revert from a putative prostate CSC to a more differentiated phenotype resembling DU145 PCa cell signature. Similar results are obtained concerning some drug resistance-related genes such as ATP Binding Cassette G2 (ABCG2) and Lung Resistant Protein (LRP). Our study provides new insights on the role of ANXA1 protein in PCa onset and progression.

## INTRODUCTION

Prostate cancer (PCa) is the prevailing cancer in US and European men and the second cause of cancer death in those populations [1]. The standard of care for PCa patients is routinely based on androgen suppression (medical or surgical castration), however, all men who undergo this treatment may develop castration-resistant prostate cancer (CRPCa) [2, 3].

The nitrogen-containing bisphosphonate (N-BP) zoledronic acid (ZA) is at present used in oncological practice to reduce skeletal related events (SREs) and pain associated to bone metastases of several cancers, including PCa. Moreover, accumulated evidences have shown that ZA may improve patient survival, reduce cancer progression and exert potent anti-tumor effects [4–6]. These anti-tumoral effects of ZA might be mainly due to its ability to inhibit farnesylpyrophosphate synthase (FPPS), a key enzyme of the mevalonate pathway that has been implicated in various aspects of carcinogenesis [7, 8].

Recent studies from our group have reported that in a ZA-resistant sub-line of DU145 PCa cells, the DU145R80 cells, continuous extensive exposure to ZA could activate the p38-MAPK pathway. This activation has a critical role in the induction of the resistance, as well as in the acquisition of a more aggressive and invasive phenotype of these cells if compared to their DU145 parental ones [9]. Moreover, in the ZA chemo-resistant DU145R80 PCa cells we identified a homogeneous group of 15 proteins differently expressed that were associated, for the most part, with regulation of cell morphology, cytoskeletal organization, cell movement and/or cell-to-cell interaction: one of these significantly deregulated proteins was identified as annexin A1 (ANXA1) [10].

ANXA1 is a 37 kDa protein able to bind (*i.e*. to annex) to cellular membranes in a Ca^2+^-dependent manner. The protein was originally reported for its anti-phospholipase activity following glucocorticoid induction however, subsequent studies from our group and others showed that ANXA1 possesses a wide range of physiological and pathological functions [11–16], some of whom correlate to cancer development [17–21].

Several studies have showed ANXA1 dysregulation in PCa. Interestingly, although overall ANXA1 expression in this tumor seems to be unaffected [22–25] or more commonly reduced [26–31], publicly available cancer microarray databases from Oncomine (http://www.oncomine.org) have shown an increase in ANXA1 expression in the more aggressive tumors [32–36].

ANXA1 biological effects could differ on varying of its intra- and extra-cellular localization [37]. Cytosolic ANXA1 for example has been frequently implicated in cytoskeletal organization since the protein binds F-actin and profilin at level of cell movement structures like lamellipodia and phillopodia, at membrane ruffles and at cell-cell contact points in several cellular models [38–39, 12]. The extracellular form of ANXA1 has been as well described to stimulate cell motility and cancer cell invasion capability, mostly interacting with specific receptors [13, 40]. These have been identified as members of the G-protein coupled formyl peptide receptor (FPRs) family that is involved for the most part in cell motility [41].

Finally, ANXA1 dysregulation has also been found to be associated with increased resistance to several anticancer drugs, including adriamycin, melphalan and etoposide, although the mechanism or mechanisms by which ANXA1 contributes to drug resistance are not fully understood, neither it is clear whether this is a general mechanism of drug resistance or is specific to particular drugs or drug classes [37, 42].

In this study, we have investigated the role of ANXA1 in the acquisition of a more aggressive phenotype in PCa cells comparing ZA-sensitive DU145 cell line with the ZA-resistant derived sub-population DU145R80. We show that in DU145R80 PCa cells ANXA1 down-regulation determines a loss of ZA-resistance, produces significant changes in cell morphology, induces a partial reversion of the Epithelial to Mesenchymal Transition (EMT), reduces the ability of these cells to spread and leads to the lack of some phenotypic features including cancer stem cell (CSC)- and drug resistance-related ones.

## RESULTS

### ANXA1 is involved in DU145R80 PCa cell resistance to ZA

By using a 2-DE DIGE proteomic approach, we show that ANXA1 is up-regulated in DU145R80 ZA-resistant PCa cells compared with their parental DU145 cells [10]. Thus, we first evaluated the role of ANXA1 in the maintenance of drug resistance to ZA in DU145R80 PCa cells.

**Figure 1:**
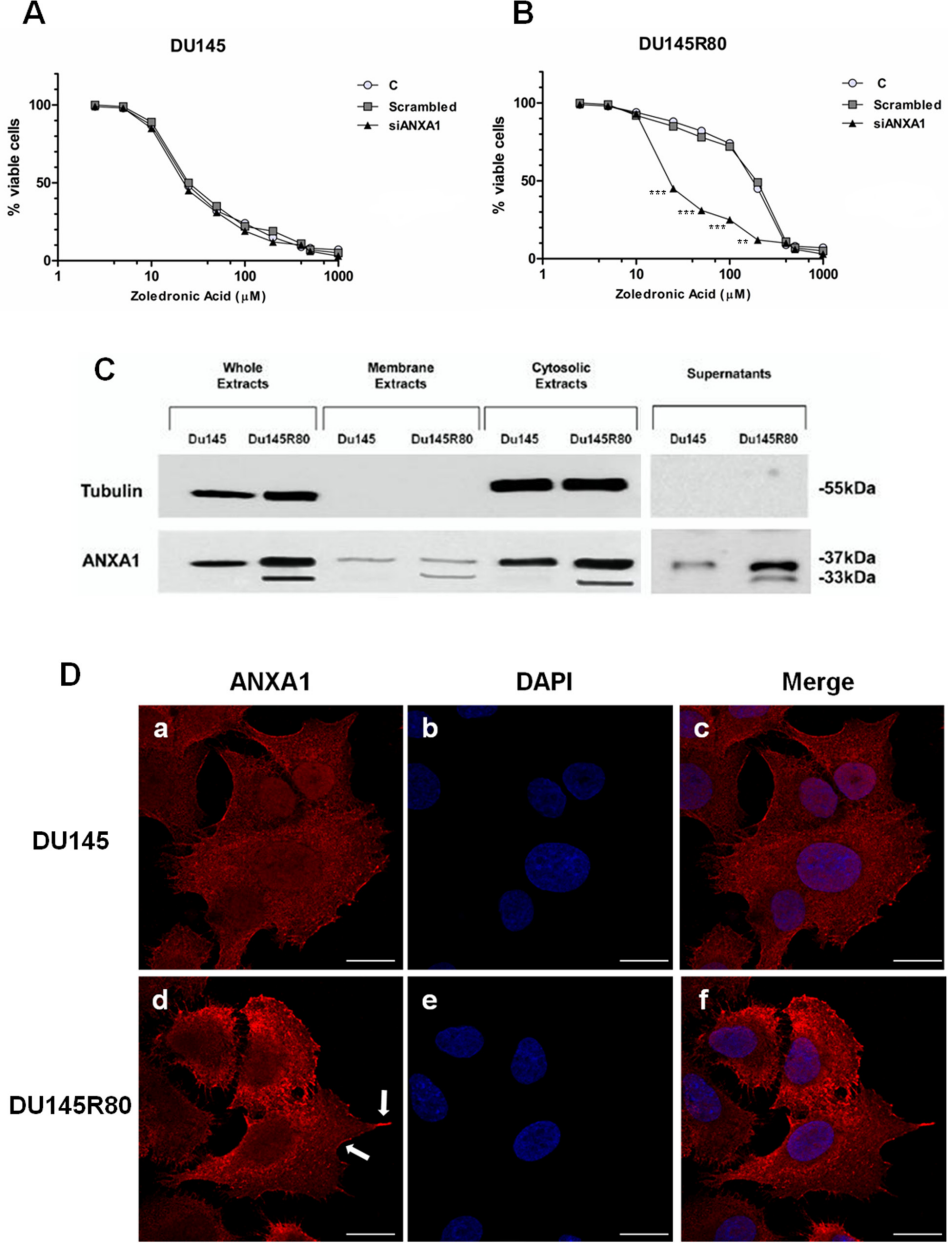
ANXA1 involvement in DU145R80 PCa cell resistance to ZA **A, B.** ZA-sensitive DU145 and ZA-resistant DU145R80 cells were treated with different concentrations of ZA (from 1 up to 200 μM) for 96 h. IC_50_ was evaluated by MTT colorimetric assay (see Materials and Methods). Absorbance relative to controls was used to determine the percentage of remaining viable cancer cells following their treatment with varying concentrations of ZA compound, which is translated to the ZA cytotoxicity and its IC_50_ values. Values are the mean ± S.E.M. from at least three independent experiments performed in triplicates (_**_p < 0.001; _***_p < 0.0001). **C.** Whole, membrane, cytosol and extracellular expression of ANXA1 in DU145 and DU145R80 cells was analyzed by Western blot with anti-ANXA1 antibody. Cellular compartments were obtained as described in Materials and Methods section. Protein normalization was performed on tubulin levels. Statistical comparisons between groups were made using one-way ANOVA or unpaired, two-tailed *t*-test comparing two variables. Differences were considered significant if *p* < 0.05 and *p* < 0.01. **D.** DU145 and DU145R80 PCa cells fixed and labeled with fluorescent antibody against ANXA1 (red). Nuclei were stained with DAPI (blue). Magnification 63x. Bar = 10 μm. Arrows indicate ANXA1 enrichment in cellular regions assigned to cell motility. All data are representative of 5 experiments with similar results.

As we showed elsewhere [9], the ZA-resistant DU145R80 cell line have a significantly higher IC_50_ compared with parental DU145 cells (109.28 ± 1.3 versus 21.3 ± 0.4, respectively; *p* < 0.0001), resulting in more than fivefold resistance to ZA (Resistance Index (RI) = 5.1) (Figure 1A, 1B). Interestingly, ANXA1 knockdown obtained by using specific siRNAs against ANXA1 (siANXA1) abolishes resistance to ZA in DU145R80 PCa cell line (IC_50_ 26.1 ± 0.97; *p* < 0.0001) (Figure 1B), suggesting that ANXA1 mediated ZA-resistance in our experimental model.

DU145R80 ZA-resistant PCa population also showed a very aggressive phenotype characterized by increased invasive capability [9].

Since extracellular occurrence of ANXA1 (cell surfaces and supernatants) has been consistently described to have several physiological and pathological functions [13, 40], we characterized ANXA1 expression and localization in sub-cellular compartments of DU145 and DU145R80 cells by 1-D Western Blotting (Figure 1C) and immunofluorescence analyses (Figure 1D, panels a–f).

Our results show that in both DU145 and DU145R80 cells ANXA1 was detectable in cytosol, membrane and extracellular compartments underlining an overall protein up-regulation in DU145R80 sub-line. Interestingly, only DU145R80 cells exhibit a strong cleavage of ANXA1, mainly in the extracellular environments (Figure 1C).

Additional analyses of ANXA1 sub-cellular localization obtained by confocal microscopy in DU145 and DU145R80 cells confirmed the membrane and cytosolic expression of ANXA1 in both cell populations and the increase of the protein in DU145R80 sub-line (Figure 1D, panels a; d). In this latter, the results highlighted ANXA1 enrichment in the cellular regions potentially assigned to cell motility, like phillopodia (Figure 1D, panel d; arrows).

### ANXA1 knockdown significantly reduced invasion capability of DU145 and ZA-resistant DU145R80 cells

Dynamic reorganization of the actin cytoskeleton leads to the development of extending protrusions in the direction of cellular motility and represents the central mechanism underlying cell invasiveness [43]. Cellular invasion can be triggered by numerous molecular signals, that are perceived by receptors on the cell surface or within cells to activate a motility response [44].

**Figure 2:**
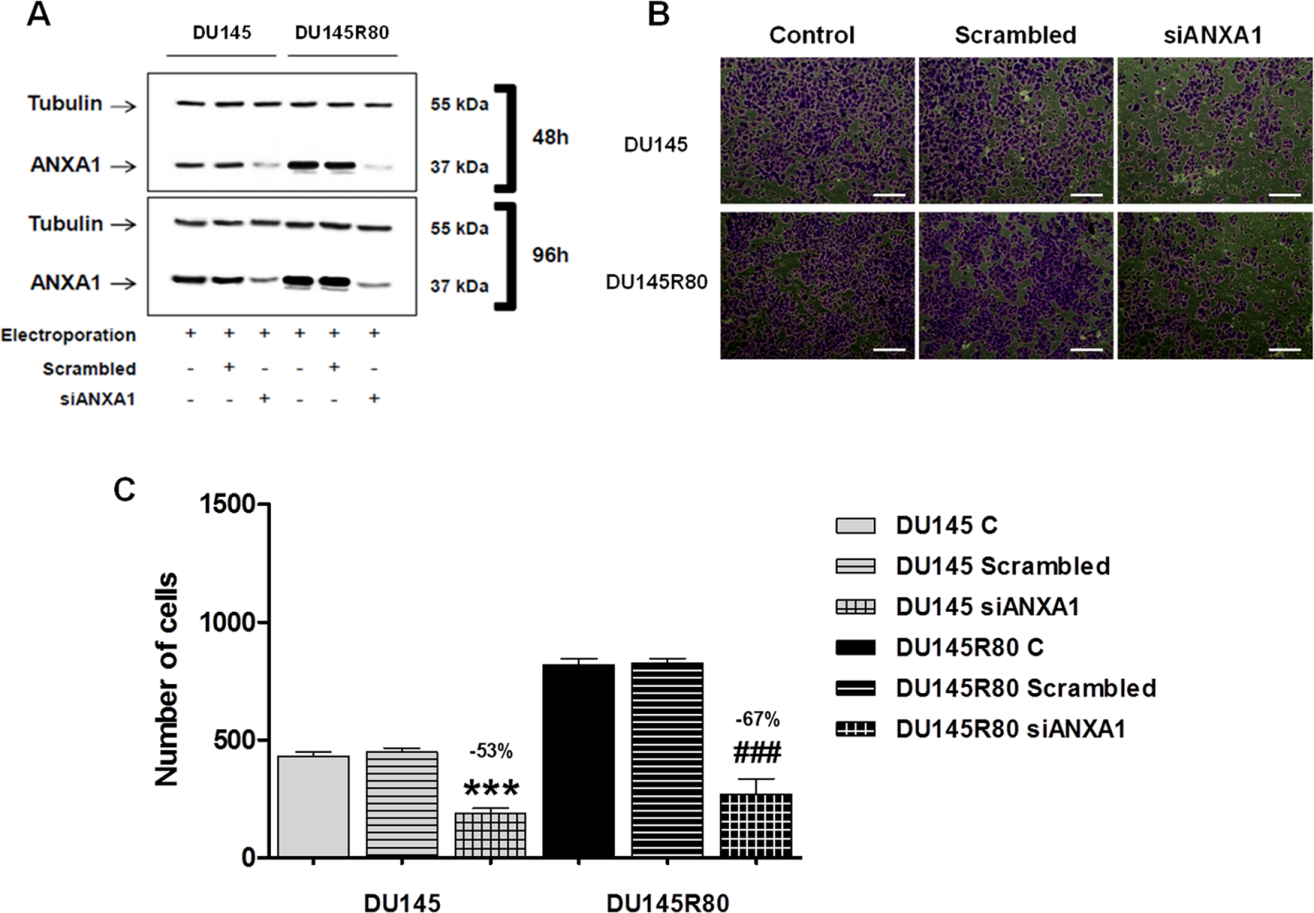
ANXA1 knockdown effects on DU145 and DU145R80 cell invasion capability **A.** Western blot using an anti-ANXA1 antibody on protein extracts from DU145 and DU145R80 cells treated or not with scrambled siRNAs (100 nM) or direct against ANXA1 (siANXA1; 100 nM) at 48 h and 96 h from transfection. 48 h Western blot corresponds to invasion assay starting point whereas 96 h refers to invasion assay ending one. Protein normalization was performed on tubulin levels. **B.** Representative bright field snapshots of invasion assay experimental end points. **C.** Invasiveness rate of DU145 and DU145R80 cells. In invasion assays a total of 90,000 cells were transfected or not with siANXA1s or scrambled siRNAs for 48 h and plated as described in Materials and Methods section. Invasiveness rate was founded out by counting stained cells on the lower surface of the filters. Data represent mean cell counts of 12 separate fields per well ± SEM of 5 experiments. ***p < 0.005 and ^###^*p* < 0.005 vs respective controls.

DU145R80 cells showed both enrichment of ANXA1 protein in cell actin-rich regions and extracellularly (cell surfaces and supernatants) and these sub-cellular localizations had been consistently described to stimulate cancer cell invasion and metastasis [17, 40]. Therefore, we next analyzed the role of ANXA1 in these processes by down-regulating the expression of the protein in DU145 and DU145R80 cells by siANXA1 (Figure 2A). As shown in Figure 2B (representative bright field pictures) and Figure 2C we confirmed, by a matrigel invasion assay, higher invasive ability of DU145R80 compared to DU145 and showed that ANXA1 knockdown markedly suppressed the invasiveness of both PCa cell lines.

### Secreted ANXA1 induces PCa cell invasion acting through FPRs in DU145 and in ZA-resistant DU145R80

Regulatory action of extracellular ANXA1 is reported to be mediated by signaling through FPRs [17, 19, 37].

**Figure 3:**
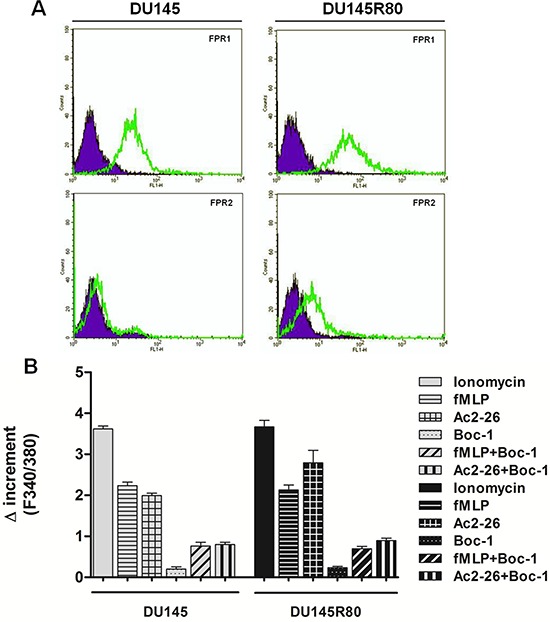
Expression and activation of FPRs in DU145 and in ZA-resistant DU145R80 **A.** Cell surface expression of FPR-1 and FPR-2 in DU145 and DU145R80 cells was analyzed by flow cytometry. The violet areas in the plots are relative to secondary antibody alone. FPR-1 and FPR-2 signals are showed with green bends. **B.** Effects of fMLP (50 nM), Ac-2-26 (1 μM) and FPR pan-antagonist Boc-1 (10 μM) on the FPR-induced intracellular Ca^2+^ increase in DU145 or DU145R80 cells. Cells were treated as described in Materials and Methods section. The histogram shows the fluorescence ratio calculated as F340/F380 nm in absence of extracellular Ca^2+^. Control represents ionomycin-stimulated cells. Data are means ± SEM (*n* = 3).

Therefore, we evaluated FPR expression in DU145 and DU145R80 cells by cytofluorimetric analysis (Figure 3A): we found that FPR-1 was similarly expressed in both cell populations whereas FPR-2 was mainly found in DU145R80 sub-line.

Several lines of evidence exist reporting that ANXA1-nFPR bond results in a series of cellular responses, such as the increase of intracellular Ca^2+^ concentration.

Differently from full length ANXA1 that only bound FPR2, the N-terminal mimetic peptide of ANXA1, Ac2-26, can activate all three human FPRs, promoting calcium fluxes, and cell locomotion [41]. Thus, we analyzed the stimulated release of Ca^2+^ from intracellular stores in DU145 and DU145R80 cells by treating cells with Ac2-26 peptide.

Cells were incubated in Ca^2+^ free medium and treated with the fluorescent calcium indicator Fluo-2AM before addition of Ac2-26 (1 μM) or the natural FPR agonist fMLP (50 nM) together or not with the FPR pharmacological antagonist Boc-1 (10 μM) that is able to antagonize all three human FPR isoforms. The spectrofluorimetric assay (Figure 3B) showed that fMLP and Ac2-26 peptide induced intracellular Ca^2+^ release in both DU145 and DU145R80 cells while no important differences between ionomycin (used as reference compound) and fMLP or Ac2-26 were detected. The effects of fMLP and Ac2-26 peptides were inhibited by the pharmacological nFPR antagonist Boc-1.

To evaluate if the pro-invasive role of ANXA1 in our PCa models is mediated by its ability to activate FPRs, as previously reported in pancreas and colon carcinomas [17, 40], we performed a matrigel invasion assay using an anti-ANXA1 blocking antibody and by stimulating or not DU145 and DU145R80 cells by administration of Ac2-26 peptide.

**Figure 4:**
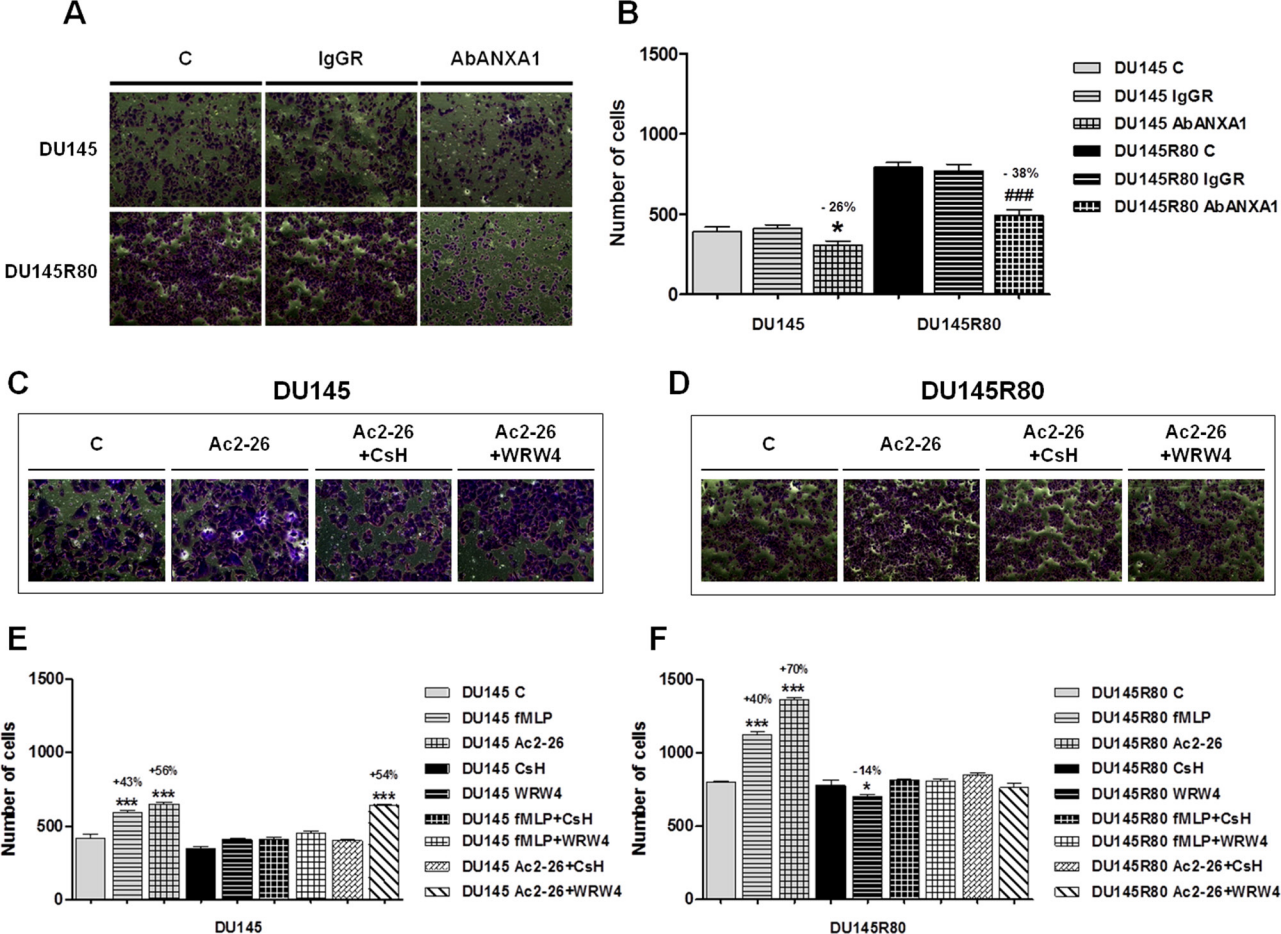
Secreted ANXA1 induces PCa cell invasion acting through FPRs **A.** Representative bright field snapshots of experimental end points of invasion assay performed by adding or not ANXA1 blocking antibody (AbANXA1) or control IgGRs. **B.** Invasiveness rate of DU145 and DU145R80 cells. Cells were treated or not with AbANXA1 or control IgGRs for 24 h and plated to perform invasion assay as described in Materials and Methods section. After 48 h, invasiveness rate was founded out by counting stained cells on the lower surface of the filters. *p < 0.05 and ^###^*p* < 0.0005 vs respective controls. **C, D.** Representative bright field snapshots of experimental end points (48 h from treatment) of invasion assay performed in DU145 and DU145R80 treated or not with Ac2-26 (1 μM), together or not with FPR-1 selective antagonist CsH (500 nM) and FPR-2 selective antagonist WRW4. **E, F.** Statistical analyses of invasion assay performed in DU145 and DU145R80 treated or not with fMLP (50 nM), Ac-2-26 (1 μM), FPR-1 selective antagonist CsH (500 nM) and FPR-2 selective antagonist WRW4 as described in Materials and Methods section. *p < 0.05 and ^***^p < 0.0005 vs respective controls.

As showed in Figures 4A (representative bright fields) and 4B ANXA1 blocking antibody was able to reduce in a significant manner DU145 and DU145R80 cell invasiveness.

Interestingly, when treated with Ac2-26 (1 μM) and fMLP (50 nM), DU145 (Figure 4C, 4E) and DU145R80 (Figure 4D, 4F) cells showed an increase in invasion through the coating of matrigel. In both of cases, experimental points were compared with non treated controls, with cells treated by the selective FPR-1 antagonist cyclosporin H (CsH; 500 nM) or the selective FPR-2 antagonist WRW4 (10 μM) (Figure 4E, 4F).

Altogether our data confirmed the functional engagement of FPR receptors by ANXA1 in regulating invasion in both DU145 and DU145R80 cells and suggested a predominant role of ANXA1/FPR-2 bond in mediating DU145R80 aggressive behavior.

### DU145R80 aggressive phenotype strictly correlated with ANXA1 expression

Cancer cells that are characterized by a more aggressive and invasive phenotype usually undergo EMT. This process drives actin polymerization and the assembly of matrix-degrading structures termed invadopodia that interface with adhesion and matrix metalloproteases (MMPs) allowing migration away from the tumor site [45].

Preceding analyses performed by Milone et al. [10] showed marked differences in cell morphology between DU145R80 and their parental DU145 cells resulting in a more invasive phenotype of the ZA-resistant sub-line.

**Figure 5:**
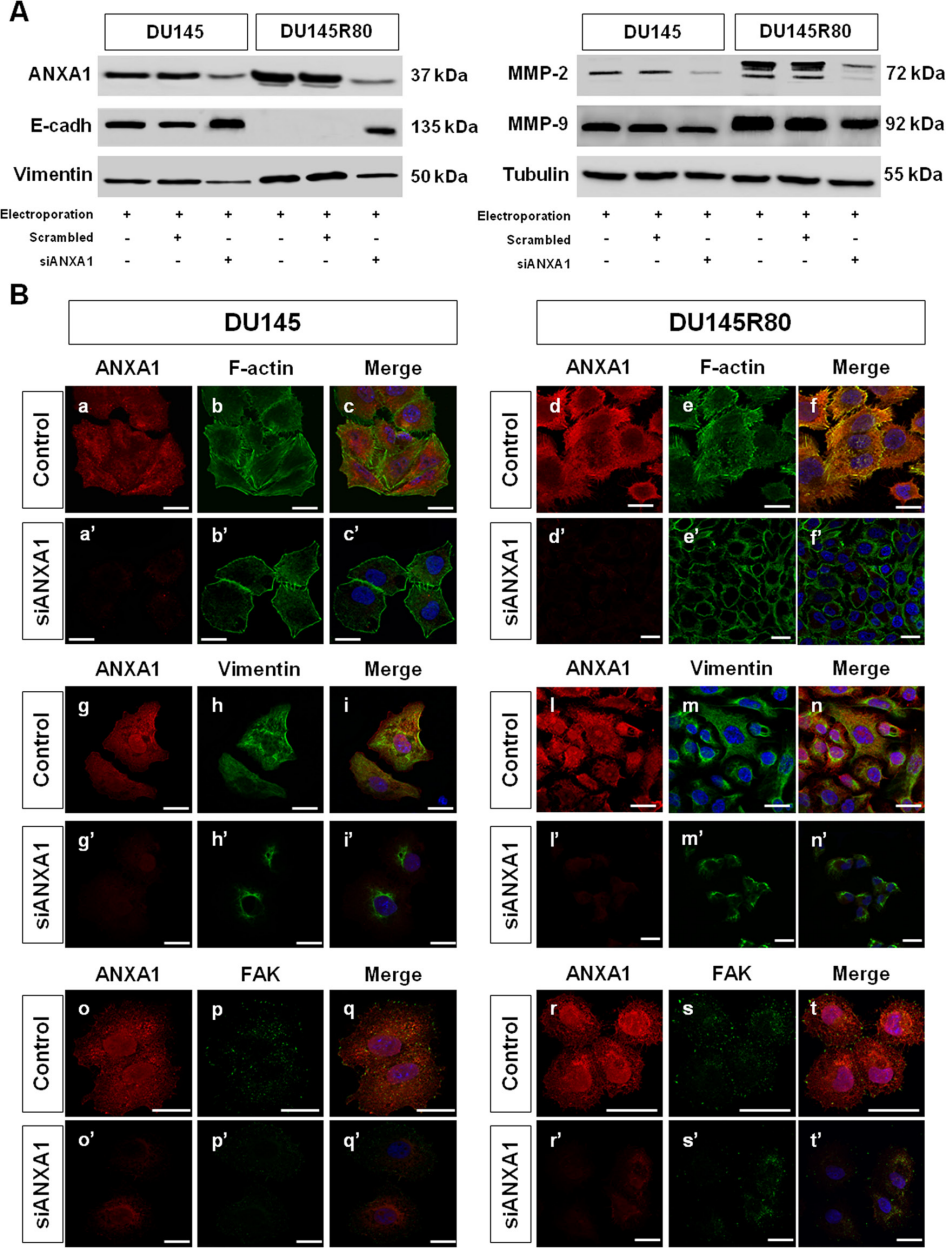
Analysis of connection between ANXA1 expression and PCa cell phenotypes **A.** Western blot using antibodies against ANXA1, E-cadherin, vimentin, MMP-2 and MMP-9 on protein extracts from DU145 and DU145R80 cells treated or not with scrambled siRNAs or siANXA1s at 48 h from transfection. Protein normalization was performed on tubulin levels. **B.** Immunofluorescence analysis to detect: F-actin in control (panels b; e) and siANXA1 treated (panels b’; e’) DU145 and DU145R80 cells, vimentin in control (panels h; m) and siANXA1 treated (panels h’; m’) DU145 and DU145R80 cells and FAK (panels p; s) and siANXA1 treated (panels p’; s’) DU145 and DU145R80 cells. ANXA1 staining was performed as transfection control (panels a, d, g, l, o, r). Nuclei were stained with DAPI. The merged images show overlapping localization of the proteins (panels c, c’, f, f’, i, i’, n, n’, q, q’, t, t’). Magnification 63x. The data are representative of 3 experiments with similar results. Bar = 10 μm.

As previously reported, ANXA1 has been frequently implicated in cytoskeletal organization and in the acquisition of cancer cell invasion as a modulator for EMT like phenotypic switch via the transforming growth factor (TGF) signaling pathway [40, 46]. Therefore, we next investigated the effects of ANXA1 knockdown on the expression of some proteins involved in EMT/invasion processes and on morphological features of DU145 and ZA-resistant DU145R80 cells, using Western blotting and confocal microscopy (Figure 5). All experiments were performed in cells treated or not with scrambled or anti-ANXA1 siRNAs, as described in Materials and Methods section.

1-D Western blotting results showed that differently from DU145, DU145R80 cells had undetectable E-cadherin and high vimentin, MMP-2 and MMP-9 expression (Figure 5A) confirming our previous observations [9, 10]. As also reported before, confocal microscopy analyses showed that DU145 cells were characterized by a more epithelial-like morphology: in these cells F-actin staining showed a well organized cytoskeleton with the appearance of several cortical stress fibers (Figure 5B, panel b; arrows), suggesting a less motile phenotype. In contrast, DU145R80 cells assumed a smaller, rounded morphology, with reduced cell-cell contact regions and the appearance of membrane condensed actin-rich structures resembling invadopodia (Figure 5B, panel e; arrows), in agreement with their reported mesenchymal features [9, 10]. Interestingly, in DU145R80 but not in DU145 cells a marked co-localization of ANXA1 with cytoskeletal actin was evident, mainly at membrane level (Figure 5B, panels a–c, d–f). Contextually, we confirmed the increase, as well as a better filamentous-like organization of vimentin in ZA-resistant DU145R80 cells (Figure 5B, panels g–i, l–n) where we also detected a high activation of focal adhesion kinase (FAK), a protein commonly expressed in adhesion hot spots of migrating/invasive cells (Figure 5B, panels o–q, r–t).

A completely different scenarios was disclosed in DU145 and DU145R80 cells when ANXA1 expression was significantly inhibited by siRNAs. In the latter conditions, an overall reversion from EMT to MET features was observed by 1-D Western blotting analyses equally in DU145 and DU145R80 cell populations. ANXA1 knockdown resulted indeed in E-cadherin increase and in vimentin, MMP-2 and -9 reduction in both PCa cell populations. Moreover, confocal microscopy observations highlighted an evident loss in cytoskeletal and vimentin organization and a marked reduction of FAK expression (Figure 5B, panels a’–f’, g’–n’, o’–t’) in siANXA1 treated cells. Altogether these data suggest a critical and hierarchical role of ANXA1 in the regulation of a mesenchymal pro-invasive phenotype in the more aggressive-ZA resistant DU145R80 cells compared to parental DU145 cells.

### ANXA1 expression correlates with CSC-like phenotype in ZA-resistant DU145R80 PCa cells

Many tumors are extensively heterogeneous by a histological point of view, with sub-populations of tumor cells characterized by distinct molecular profiles [47]. Tumors may also include CSCs, rare cancer cells (generally <1% on total cells in a tumor mass) with indefinite potential for self-renewal that drive tumorigenesis. To date, the possible existence of CSCs has been identified in several solid malignancies including PCa, revealing the critical role of these cells in metastasis and drug resistance [48, 49].

Our experimental observations suggested that DU145R80 cells display two features of CSCs lacking in their parental counterparts such as increased invasive capability and EMT. Therefore, we investigated if DU145R80 cells were enriched for CSCs, using a number of approaches.

**Figure 6:**
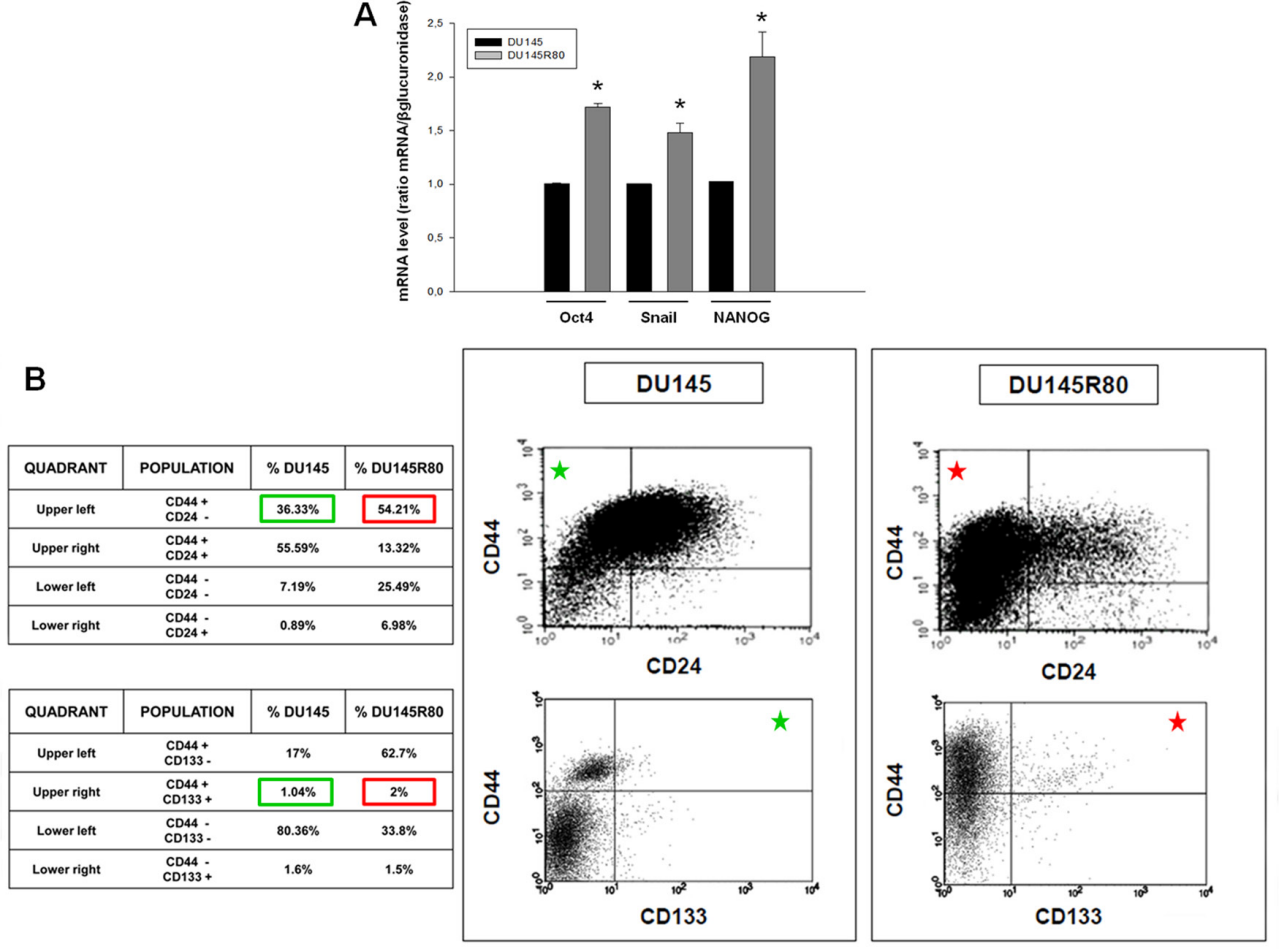
Evaluation of stem cell markers by Real-time PCR and flow cytometry in ZA-sensitive DU145 and ZA-resistant DU145R80 cells **A.** Oct4, NANOG and Snail mRNA expression was evaluated by Real-time PCR after 24 h of cell culture. The data are representative of at least three independent experiments, include the means ± SD of technical triplicates and reported statistical analysis of DU145R80 versus DU145 cells (*p = 0.001 Oct4; *p* = 0, 016 Snail; *p* = 0, 019 NANOG). **B.** Scatter plots of CD44/CD24 and CD44/CD133 expression for DU145 compared with DU145R80 cells. Green squares in the table referred to DU145 CD44^high^/CD24^low^ and CD44^high^/CD133^high^ populations also marked with green stars in the respective quadrants of dot plots. Red squares and stars referred to DU145R80 populations.

At first, as shown in Figure 6A we demonstrated in DU145R80 a clear mRNA over expression of Oct4, NANOG and Snail, all markers characteristic of PCa cancer stem cells [50, 51].

Next, we showed by flow cytofluorimetric analyses an enrichment of CD44^high^/CD24^low^ and CD44^high^/CD133^high^ cells which are considered a distinct sub-population of early progenitor/SCs in PCa tumors, in DU145R80 (Figure 6B, red squares) compared to DU145 cells (Figure 6B, green squares) [52].

We next investigate possible effects of ANXA1 knockdown on CSC signature in both DU145 and aggressive DU145R80 PCa cell lines.

**Figure 7:**
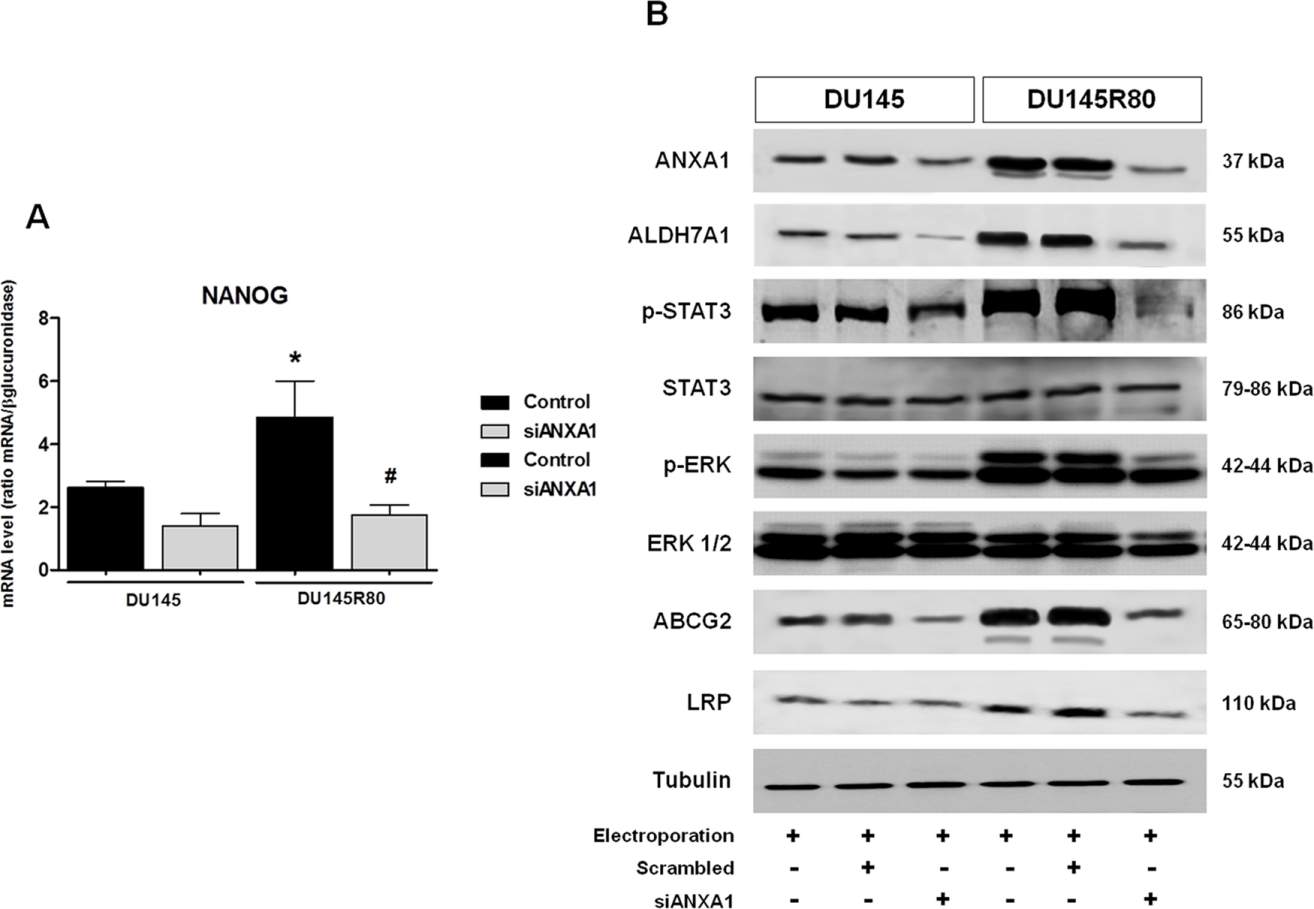
ANXA1 expression correlates with CSC-like phenotype in ZA-resistant DU145R80 PCa cells **A.** NANOG mRNA expression was evaluated by Real-time PCR at 48 h from transfection in control and siANXA1 treated DU145 and DU145R80 cells. The data are representative of at least three independent experiments, include the means ± SD of technical triplicates and reported statistical analysis of DU145R80 versus DU145 cells. *p < 0.05 DU145R80 control vs DU145 control; ^#^*p* < 0.05 siANXA1 DU145R80 vs DU145R80 control. **B.** Western blot using antibodies against ANXA1, ALDH7A1, p-STAT3, STAT3, p-ERK, ERK 1/2, ABCG2 and LRP on protein extracts from DU145 and DU145R80 cells treated or not with scrambled siRNAs or siANXA1s at 48 h from transfection. Protein normalization was performed on tubulin levels. The data are representative of 3 experiments with similar results.

Thus, we analyzed mRNA levels of Oct4, NANOG and Snail in both upon ANXA1 knockdown. Interestingly, 48 h following transfection with siANXA1 we observed a significant reduction of NANOG mRNA levels in both DU145 and DU145R80 ANXA1 knockdown cells (Figure 7A) whereas no considerable differences were observed in Oct4 and Snail transcription levels (data not shown).

High ALDH7A1 that we have previously demonstrated to be up-regulated in DU145R80 cells compared to DU145 [10], together with CD44^high^/CD24^low^, NANOG and Oct4 expression has been reported by several studies to identify CSC phenotype in human PCa [53–59]. In fact, ALDH7A1 affects a number of genes and factors involved in migration, invasion and metastasis, including transcription factors such as Snail1/2 and can be used to identify tumor-initiating and metastasis-initiating cells in various human carcinomas, including PCa. Notably, we confirmed the up-regulated ALDH7A1 in DU145R80 cells (Figure 7B) compared to parental DU145 cells and showed that ANXA1 knockdown was able to reduce its expression only in ZA-resistant PCa sub-line (Figure 7B).

Hereafter, we performed 1-D Western blot analyses of expression profiles of some protein involved in different ways in CSC signature, gain and maintenance and/or related drug resistance capability.

The connection between JAK2/STAT3 pathway activation and CSCs has been highlighted in a previous work on ovarian cancer, where the SC marker CD44 together with the embryonic SC marker NANOG have been associated with the activation of STAT3 [60]. As expected, in ZA-resistant DU145R80 cells we found a higher STAT3 phosphorylation compared with their parental DU145 cells that to the contrary was significantly reduced in DU145R80 ANXA1 knockdown cells (Figure 7B).

Subsequently, as MAPK/ERK signaling pathway has been linked to metastasis [61], EMT [62], and to cancer SC/tumor initiating cells [63], we analyzed by 1-D Western blot the phosphorylation of extracellular signal-regulated kinase (ERK) which is a measure of activated ERK, in not treated and ANXA1 knockdown PCa cells. Our results showed that only DU145R80 had high activation level of ERK that again was strongly reduced in siANXA1 treated cells (Figure 7B).

It is well documented that CSCs express high levels of drug resistance proteins that explain side-population features. The ATP-binding cassette (ABC) transporter proteins represent the largest family of trans-membrane proteins to confer drug resistance to tumor cells, and in particular, ABC transporter-subfamily B member 1 (ABCB1/MDR1/P-glycoprotein, P-gp), subfamily C member 1 (ABCC1/MRP1) and subfamily G member 2 (ABCG2/BCRP) are considered to be the most important transporters [64]. Additional proteins that seems to be important in stimulating the development of multidrug resistance in cancer cells are the Major Vault Proteins (MVP) and, in particular, the Lung Resistance Protein (LRP) [65]. Repeated immunoblotting analysis indicated consistent differences between DU145 and DU145R80 in the expression of ABCG2/BCRP, while apparently neither of the cell lines expressed MDR1 or MRP1 (data not shown). In addition, DU154R80 cells showed a clear over-expression of LRP compared to DU145 cells (Figure 7B). Interestingly, ANXA1 knockdown reduces this protein expression only in ZA-resistant sub-line whereas their amount appeared to be unaffected in DU145 parental cell line (Figure 7B). Altogether these data suggest a critical role of ANXA1 in regulating CSC phenotype in our PCa models.

## DISCUSSION

The cell-origin theory for intratumoral heterogeneity proposes that tumor initiation from distinct cell types in the lineage hierarchy gives rise to tumor subtypes with different prognoses and/or treatment responses [66, 67]. In PCa, this model has not been methodically investigated as for other tumor types, although several reports investigated PCa putative stem cell-like cells [48, 50, 52, 53, 55–59]. PCa is composed of phenotypically exocrine, luminal, and dispersed neuroendocrine cells. It was long believed that this tumor lacked basal cells, as many investigators had shown the absence of basal cell keratins and that cancer initiating cells were therefore of luminal origin [68–70].

However, relatively recent evidences from the mouse suggested the existence of a third epithelial cell type derived from the basal layer, identified by expression of both the “basal” keratin K5 together with the “luminal” keratin K18 and therefore known as “intermediate” population. The identification in PCa primary tumors of these transiently proliferating/amplifying cells which are intermediate to SCs and fully differentiated cells [71] implicate both luminal [72] and basal cells [73–75] in PCa initiation. This has been involved in CRPCa and drug-resistance insurgency as expansion and differentiation of these transiently proliferating/amplifying cells during androgen deprivation therapy might subsequently lead to androgen-independent progression of PCa [75, 76, 53].

The association of intermediate cells with CRCP was also reflected in cell lines. While the androgen-dependent cell line LNCaP expressed only K18, the androgen-independent cell lines PC3 and DU145 express both K5 and K18 [76]. Interestingly, LNCaP cells are characterized by low expression of ANXA1 whereas considerable expression of ANXA1 is reported for PC3 and DU145 cell lines [77].

The role of ANXA1 in cancer progression is still discussed as this protein may have specific functions in different tumoral models.

ANXA1 protein is mainly described to be reduced in PCa [26–31, 78, 79]. Nevertheless, conflicting datasets exist that suggest ANXA1 over-expression in this tumor [32–36]. In normal prostate tissue ANXA1 expression seems to be confined in basal cells and these latter are extremely rare in PCa mass [80], thus it is likely that the inconsistent results that were reported may arise from misunderstanding interpretation on whole biopsies due to the lacking of a cell-specific identification of ANXA1 expression in the heterogeneity of the tumoral mass.

Interestingly, we have recently reported ANXA1 up-regulation in a ZA-resistant very aggressive sub-line derived from DU145 PCa cells [9, 10].

In the present study we show in this syngenic models that ANXA1 is involved in the maintenance of some phenotypic features including CSC- and drug resistance-related ones.

Our results show that in both DU145 and DU145R80 cells ANXA1 was detectable in cytosol, membrane and supernatants underlining a whole protein over-expression in DU145R80 sub-line, in which a strong cleavage of ANXA1, mainly in extracellular environments was also observed.

ANXA1 could be exported extracellularly by the ATP binding cassette A1 (ABC-A1) transporter system [81, 82] which is highly expressed in androgen-independent PCa cell lines [83]. In this regard we have shown that DU145R80 cells over-express ABCG2/BCRP transporter. However, further studies are needed to address these points.

Analyses of ANXA1 sub-cellular localization also highlighted an interesting protein enrichment in the cellular regions involved in cell motility in DU145R80 ZA-resistant sub-line. Comparison between DU145R80 with their parental DU145 cell invasion capability, by knocking down ANXA1 expression, showed that the reduction of the protein markedly suppressed the invasiveness of both PCa cell populations. Since we also observed ANXA1 appearance in PCa cell supernatants, the effects of the protein on cell invasion capability could be arguably carried out in two different ways, as occurs in other physiological and pathological systems [15, 40].

First, ANXA1 could participate in actin reorganization by altering cell adhesion and increasing the formation of cell membrane protrusions through direct bond with actin [60–62, 24]; second, ANXA1 could extracellularly bind FPRs which are reported to lead to cell motility by inducing cytoskeletal reorganization. We found that DU145 and DU145R80 cells both express FPR-1 whereas FPR-2 was predominantly detected in DU145R80.

FPR-2 rather than FPR-1, has been involved in tumor progression and metastases of several tumors [84–88]. A variety of agonists in several cell types efficiently binds FPR-2 and these bonds lead to the activation of intracellular signaling molecules including STAT3, PLC-γ1/PKCα and PI3K/Akt pathways [89] and mitogen-activated protein kinases (MAPK), such as p38MAPK [90, 91] that interestingly is up-regulated in DU145R80 compared to DU145 cells [9]. Moreover, FPR-2 is high expressed in basal rather than in luminal breast cancer cells [86] and it was very recently implicated in pluripotency associated gene expression, self-renewal, invasion and tumorigenicity of CSCs from pancreatic ductal carcinoma (PDAC) [92] suggesting a crucial role of the receptor in the less differentiated and therefore more aggressive tumors.

Our observations about the marked FPR-2 expression in the more aggressive DU145R80 sub-line could be thus in line with those previously reported observations. Moreover hyper-activation of both STAT3 and ERKs in DU145R80 cells reinforce our hypothesis.

Different expression of FPRs in ZA-sensitive and ZA-resistant PCa cells was also reflected in the experiments we performed to analyze the effects of extracellular ANXA1 on PCa cell invasion capability. In fact, while the administration of ANXA1 blocking antibody and fMLP equally affected DU145 and DU145R80 cell motility by reducing or increasing invasiveness respectively, Ac2-26 peptide that has higher affinity for FPR-2 if compared to full length ANXA1 [93], appeared to be more effective in DU145R80 sub-line. Accordingly, the FPR-2 selective antagonist WRW4 alone was able to partially reduce DU145R80 cell invasiveness whereas had no effect in DU145 cells.

PCa cell ability to maintain some flexibility results in the aptitude to gain a more aggressive behavior that is a critical factor for development of CRPCa advanced disease. In epithelial cancers, this plasticity may involve, at least in part, the EMT and the reverse process MET.

We have previously reported that, compared to parental DU145, DU145R80 cells demonstrated resistance to apoptosis and anoikis, over-expression of anti-apoptotic and oncogenic proteins, EMT and increased expression of the metalloproteases 2 and 9 [9].

Since ANXA1 protein has been reported to promote migration and invasion of metastatic basal-like breast cancer cells as a modulator for EMT phenotypic switch through the transforming growth factor (TGF) signaling pathway [46], our hypothesis was that ANXA1 could induce the acquisition of a mesenchymal phenotype in our PCa cell models.

As expected, in ANXA1 knockdown conditions we observed a broad reversion from a EMT to a MET phenotype, similarly in DU145 and DU145R80 cell populations. This was characterized by E-cadherin increase and MMP-2 and -9 reduction. Moreover, ANXA1 decrease led to a marked failure in cytoskeletal and vimentin organization and a striking reduction of FAK expression.

Interestingly, a role for ANXA1 was identified during embryonic development and the proliferation-dependent processes of normal *versus* cancer cell differentiation [20, 13] although few evidences were reported about ANXA1 role in SC as well as in CSC biology [12, 94].

CSCs and metastatic cells share some features, such as EMT and invasion capability so that CSCs have been reported as responsible for migration from the site of the primary tumor and thus starting metastases [95, 96].

Our data showed that DU145R80 sub-population exhibits an increased capability to undergo basement membrane invasion and a marked EMT phenotype compared to DU145 parental PCa cell line.

Several markers are reported to identify prostate CSCs including CD44, CD133 [97], NANOG [50] and the stemness-associated gene products Snail, Sox2 and Oct4 [51]. Cells containing all, or some, of these markers are a lot more tumorigenic when compared to the complete, non fractionated tumor cell populations [52, 55, 56].

Notably, we showed that DU145R80 cells have increased mRNA levels for NANOG, Snail and Oct4, are CD44^high^ and exhibit gene expression profiles consistent with those of CD44^high^CD24^low^/CD44^high^CD133^high^.

CSCs from PCa show resistance to chemotherapy and radiotherapy [45] therefore, it is likely that the selective pressure of drugs used during CRPCa treatment also induces PCa cells to acquire features of CSCs, engendering treatment resistance. Accordingly, after castration a recurrent increase of stem cell-like features is observed in mice and CSC marker expression also increases in basal PCa cells after androgen deprivation therapy [98].

It has been reported that ANXA1 from prostate-derived cancer-associated fibroblasts (CAF) is capable of inducing EMT, promoting *de novo* generation of CSCs and stimulating the CSC population from PCa cells [94]. Here, we demonstrate that ANXA1 expression in DU145R80 PCa cells correlates with several genes involved in the acquisition/maintenance of a CSCs phenotype and/or drug resistance such as NANOG [50], ALDH1A7 [54], STAT3 [99–102], ERK [61–63], ABCG2 [64] and LRP [65].

The classical mechanism of tumor associated drug-resistance mainly includes the expression of various resistant genes, proteins and enzymes, as well as the regulation of relevant signal transduction pathways [103]. A correlation between ANXA1 expression and drug resistance was observed in different tumors where the protein seems to act by inducing the drug resistance behavior in lung adenocarcinoma [104], pancreatic cancer [105] and ovarian cancer [106] or by reducing it in bladder cancer [107], hepatoma [108] and myeloid leukemia [109].

In conclusion, on the basis of our findings we suggest that ANXA1/FPR-2 bond could activate Jak/STAT3 and ERK1/2 pathways and initiate the phosphorylation of tyrosine residues, translocation of STAT dimer and activation of transcription [89]. This may cause the up-regulation of E-cadherin repressors and alter the polarity of tumor epithelium. All these pathways may facilitate the EMT and the status of drug resistance likely leading to the acquisition/maintenance of CSCs signature to some extent. Therefore, ANXA1 may be considered as a novel candidate marker to identify aggressive PCa phenotypes and/or to represent a novel target for therapy in advanced disease. Clearly, the temporal and spatial details of changes in expression of ANXA1 in PCa tumors remain to be clearly defined and further studies are needed to address this point.

## MATERIALS AND METHODS

### Cell culture and ZA-resistant cell selection

The PCa cell line DU145 was purchased from American Type Culture Collection (Rockville, MD, USA). ZA-resistant DU145R80 cells were obtained by treating DU145 with increasing concentrations of ZA as previously described [9]. DU145 and DU145R80 cells were grown in RPMI 1640 (Lonza) containing 10% of heat-inactivated fetal bovine serum (FBS; Lonza), 10000 U/ml penicillin and 10 mg/ml streptomycin (Lonza), 20 mM Hepes (pH 7.4) and 4 mM L-glutamine. The cells were grown in a humidified atmosphere composed of 95% air and 5% CO_2_ at 37°C. Suspension culture was performed in Ultra-low attachment flasks (Corning Incorporated Life Sciences, Tewksbury, MA, USA).

### siRNA transfection

The knockdown of ANXA1 in DU145 and DU145R80 cells was performed using siRNAs. siRNAs targeting human ANXA1 were purchased from IDT (Integrated DNA Technologies Inc., Coralville, IA, USA). The sequences to target ANXA1 were: sense 5′-ATG CCT CAC AGC TAT CGT GAA -3′ and anti-sense 5′- TTC ACG ATA GCT GTG AGG CAT -3′. siRNA Oligo-Scrambled (Santa Cruz Biotechnology) was used as control at the same concentration. PCa cells were initially plated in media containing 10% FBS. After 24 hours, cells were washed once with PBS and transfected or not with siRNAs by Nucleofector (Lonza) according to the manufacturer's instructions. The cells were processed for Western blot analysis and confocal microscopy at 24, 48, 72 and 96 hours after transfection. Invasion assay experiments were performed at 48 hours from transfection. siRNA treated cells were harvested at 48 hours from transfection for RT-PCR analyses.

### MTT assay

ZA in sterile H_2_O (from 1 up to 200 μM) was administered to PCa cells and IC_50_ was evaluated in DU145 and DU145R80 cell lines by MTT assay, as previously described [110]. Briefly, DU145 and DU145R80 cells were seeded at 15 × 10^3^ cells/well in a 96-well plate and incubated for the indicated times (48, 72 and 96 hours) at 37°C.

At the ends of the selected experimental times, MTT stock solution (5 mg/ml) was added to all wells of an assay (25 μl per 100 μl medium), and plates were incubated at 37°C for 4 hours. At the end of each experimental point, cells were lysed and the dark blue crystals dissolved with 100 μl of a solution containing 50% (v/v) N, N-dimethylformamide, 20% (w/v) SDS with an adjusted pH of 4.5. The optical density (OD) of each well was measured with a microplate spectrophotometer (Titertek Multiskan MCC/340) equipped with a 620 nm filter. The viability of cells in response to treatment with tested compounds was calculated as: % viable cells = [OD (550 nm-690 nm) ZA/OD (550 nm-690 nm) negative control] × 100.

ZA IC_50_ was determined using Prism 5.0 (GraphPad Software Inc.). IC_50_ values were presented as means ± SEM of at least three independent experiments carried out by triplicate.

### Cytosol and membrane extracts

DU145 and DU145R80 cells were washed twice with PBS, detached with trypsin-EDTA 1x in PBS (Euroclone), harvested in PBS and centrifuged for 5 minutes at 600 × g at 4°C. After that, cells were lysed in 4 ml of buffer A (Tris HCl 20 mM, pH 7, 4; sucrose 250 mM; DTT 1 mM; protease inhibitors, EDTA 1 mM in water), sonicated (5 seconds pulse - 9 seconds pause for 2 minutes, amplitude 42%) and then centrifuged at 4°C for 10 minutes, at 5000 × g. The resulting supernatants were ultra-centrifuged for 1 hour at 100000 × g at 4°C, until to obtain new supernatants corresponding to cytosol extracts. Each resultant pellet was dissolved in 4 ml of buffer A and ultra-centrifuged for 1 hour at 100000 × g at 4°C. The pellets were then resuspended in 250 μl of buffer B (Tris HCl 20 mM, pH 7, 4; DTT 1 mM; EDTA 1 mM; Triton X-100 1%, in water) and left overnight on orbital shaker at 4°C. Next, the solution was centrifuged for 30 minutes at 50000 × g at 4°C: the supernatants represent membrane extracts.

### Supernatant analysis

Cell growth media were harvested, frozen at −80°C and lyophilized. Dried samples were suspended in lysis buffer containing protease inhibitors and left at 4°C for 30 minutes. After centrifugation, the supernatants were filtered through Amicon Ultra-15, PLTK Ultracel-PL Membrane, 10 kDa (Millipore).

### Western blotting analysis

Protein expression was examined by SDS-PAGE. Total intracellular proteins were extracted from the cells by freeze/thawing in lysis buffer containing protease inhibitors. Protein content was estimated according to Biorad protein assay (BIO-RAD). Samples (20 μg protein) were loaded onto denaturing-polyacrylamide gel and separated by SDS-PAGE. The separated proteins were then transferred electrophoretically to nitrocellulose membranes (Immobilon-NC, Millipore). Membranes were blocked with 5% non-fat dry milk in TBS-Tween 20 (0.1% v/v) and then incubated overnight at 4°C with the primary antibodies. Proteins were visualized using the enhanced chemioluminescence detection system (Amersham Pharmacia Biotech) after incubation overnight at 4°C with primary antibodies as follow: ANXA1 (rabbit polyclonal; 1:10000; Invitrogen), E-cadherin (goat polyclonal; 1:500, Santa Cruz Biotechnology), vimentin (mouse monoclonal; 1:5000; Santa Cruz Biotechnology), MMP-2 (rabbit monoclonal; 1:1000; Abcam), MMP-9 (rabbit polyclonal; 1:1000; Abcam), STAT3 (rabbit monoclonal; 1:1000; Cell Signaling), p-STAT3 (rabbit polyclonal; 1:1000; Cell Signaling), ERK 1/2 (rabbit polyclonal; 1:1000; Cell Signaling), p-ERK 1/2 (rabbit polyclonal; 1:1000; Cell Signaling), ALDH7A1 (rabbit monoclonal; 1:5000; Abcam), ABCG2 (rabbit polyclonal; 1:1000; Cell Signaling), LRP (mouse monoclonal; 1:200; Santa Cruz Biotechnology) and monoclonal a-tubulin (1:5000; Sigma-Aldrich). Membranes were then incubated at room temperature with an appropriate secondary rabbit, mouse or goat antibody (1:5000; Sigma-Aldrich). Immunoreactive protein bands were detected by chemioluminescence using enhanced chemioluminescence reagents (ECL; Amersham), the blots were exposed and analyzed to Las4000 (GE Healthcare Life Sciences).

### Confocal microscopy

After the specific time of incubation, DU145 and DU145R80 cells were fixed in p-formaldehyde (4% v/v in PBS) for 5 minutes. The cells were permeabilized in Triton X-100 (0.5% v/v in PBS) for 5 minutes, and then incubated in goat or donkey serum (20% v/v PBS) for 30 minutes, and with primary antibodies against ANXA1 (rabbit polyclonal; 1:100; Invitrogen), vimentin (mouse monoclonal; 1:500; Santa Cruz Biotechnology) and FAK (mouse monoclonal; 1:100; BD Transduction Laboratories), overnight at 4°C. After two washing steps with PBS, cells were incubated with anti-rabbit and / or anti-mouse AlexaFluor (488 and/or 555; 1:1000; Molecular Probes) for 2 hours at RT and then with FITC-conjugated anti-F-actin (5 μg/ml; Phalloidin-FITC, Sigma) for 30 minutes at RT in the dark. Hoechst 33342 (Molecular Probes) was used to detect nuclei. The coverslips were mounted in Mowiol (Mowiol 4–88, Sigma-Aldrich). A Zeiss LSM 710 Laser Scanning Microscope (Carl Zeiss MicroImaging GmbH) was used for data acquisition. Images were generated with Zeiss ZEN Confocal Software (Carl Zeiss MicroImaging GmbH).

### Invasion assay

DU145 and DU145R80 invasion assay was performed as previously described with minor modifications [63]. The role of ANXA1 on PCa cell invasiveness was analyzed in control conditions, by transfecting the cells with scrambled siRNAs or siANXA1s, by administration of an ANXA1 blocking antibody (AbANXA1) or control IgGRs, or by administration of nFPRs agonists/antagonists. The analysis of the effects produced on cell invasion was carried out after 48 h from treatments. Administration of nFPR agonists/antagonists to PCa cells was performed as follows: fMLP (50 nM), Ac2-26 (1 μM), Boc-1 (10 μM), ciclosporin H (CsH; 500 nM), WRW4 (10 μM). The number of cells that had migrated to the lower surface was counted in twelve random fields using EVOS light microscope (10X) (Life technologies Corporation). The experiments were performed in triplicate.

### Flow cytometry

DU145 and DU145R80 cells were harvested at a number of 1 × 10^6^ and centrifuged at 30000 × g for 5 minutes. The pellets were then incubated on ice for 1 hour in 100 μl of PBS containing a primary polyclonal antibody against FPR-1 (1:500, Santa Cruz Biotechnology), a primary monoclonal antibody against FPR-2 (1:100, Genovac), a primary APC-conjugated antibody against CD44 (2 mg/ml), a primary monoclonal PE-conjugated antibody against CD24 (2 mg/ml) and a primary monoclonal PE-conjugated antibody against CD133 (1 mg/ml) (Miltenyi Biotec, Calderara di Reno, Bologna, Italy). For FPR-1 and -2 staining DU145 and DU145R80 cells were next washed twice and incubated on ice for 1 hour in 100 μl of PBS containing AlexaFluor 488 anti-rabbit (1:1000; Molecular Probes) or Alexa-Fluor 488 anti-mouse (1:1000; Molecular Probes). The cells were analyzed with Becton Dickinson FACScan flow cytometer using the Cells Quest program.

### Measurement of intracellular Ca^2+^ signaling

Intracellular Ca^2+^ concentrations [Ca^2+^] were measured using the fluorescent indicator dye Fura 2-AM, the membrane-permeant acetoxymethyl ester form of Fura 2, as previously described [63], with minor revisions. Treatment with ionomycin (1 μM, Sigma Aldrich), fMLP (50 nM, Sigma Aldrich), with Ac2-26 (1 μM, Tocris Bioscience) or Boc-1 (10 μM, Bachem AG) was performed by adding the appropriate concentrations of each substance into the cuvette. The excitation wavelength was alternated between 340 and 380 nm, and emission fluorescence was recorded at 515 nm. The ratio of fluorescence intensity of 340/380 nm (F340/F380) was used to estimate intracellular free calcium. Results are indicated as delta increase of fluorescence ratio (F340/F380 nm) induced by ionomycin-basal fluorescence ratio (F340/F380 nm).

### RNA isolation and quantitative RT-PCR assay

Real-time PCR (RT-PCR) was performed as described by Milone et al. [9]. Briefly, total RNA was isolated using the RNeasy plus mini kit (Qiagen, Hilden, Germany) as indicated by the manufacturer's instructions and quantified using a NanoVue Plus spectrophotometer (GE Healthcare). Reverse transcription was performed using the QuantiTect Reverse Transcription Kit (Qiagen). QuantiTect Primer Assays (Qiagen) were used to quantify RNA levels of NANOG (*Hs NANOG 1 SG*, NM_024865), Snail (*Hs SNAI1 1 SG*, NM_005985), GUSB (GUSB Forward AGCCAGTTCCTCATCAATGG, GUSB Reverse GGTAGTGGCTGGTACGGAAA) and Oct4 (Oct4 Forward TGGGATATACACAGGCCGATG, Oct4 Reverse TCCTCCACCCACTTCTGCAG). Each sample was assayed in quadruplicate with 20 ng of input RNA per well in a 25 μl reaction volume containing 1_QuantiTect SYBR Green PCR Master Mix and 1_QuantiTect gene expression assay (Qiagen). The specificity of the produced amplification product was confirmed by examination of dissociation reaction plots. Cycle threshold values (Ct) generated using Sequence Detection System 2.2.2 (Applied Biosystems, Foster City, CA, USA) default parameters were exported to determine relative mRNA abundances among genes in the classifier.

All gene expression levels were normalized to GUSB expression. Each sample was tested in triplicate using RT-PCR and the ABI Prism 7900 HT Sequence Detection System (Applied Biosystems), and three independent experiments were used to quantify relative gene expression.

### Statistical analysis

Representative results from Western blots from a single experiment are presented; additional experiments yielded similar results. The optical density of the protein bands detected by Western blotting was normalized against tubulin levels. Statistical comparisons between groups were made using one-way ANOVA or unpaired, two-tailed *t*-test comparing two variables. Differences were considered significant if *p* < 0.05 and *p* < 0.01. The results of invasion assays are expressed as the means for at least five independent experiments (± S.E.M.). The RT-PCR data for mRNA expression are representative of at least three independent experiments and include the means ± S.E.M. of technical triplicates. Statistical significance was proved by two-sided Student's *t*-tests (normal distribution), and all statistically significant *p*-values (≤0.05) are reported in the manuscript or in figure legends.
